# The impact of postoperative inclination of the joint line on clinical outcomes in total knee arthroplasty using a prosthesis with anatomical geometry

**DOI:** 10.1038/s41598-023-28182-2

**Published:** 2023-01-18

**Authors:** Manabu Yamada, Arata Nakajima, Masato Sonobe, Yorikazu Akatsu, Keiichiro Yamamoto, Junya Saito, Masaki Norimoto, Keita Koyama, Shinji Taniguchi, Yasuchika Aoki, Toru Suguro, Koichi Nakagawa

**Affiliations:** 1 Department of Orthopaedic Surgery, Toho University Graduate School of Medicine, 5-21-16 Omori-nishi, Ota-ku, Tokyo, 143-8540 Japan; 2 Department of Orthopaedic Surgery, Toho University Sakura Medical Center, 564-1 Shimoshizu, Sakura, Chiba 285-8741 Japan; 3 Department of Orthopaedic Surgery and Rehabilitation, Toho University Sakura Medical Center, 564-1 Shimoshizu, Sakura, Chiba 285-8741 Japan; 4 Department of Orthopaedic Surgery, Eastern Chiba Medical Center, 3-6-2 Okayamadai, Togane, Chiba 283-8686 Japan; 5 Department of General Medical Sciences, Graduate School of Medicine, Chiba University, 1-8-1 Inohana, Chuo-Ku, Chiba 260-8677 Japan; 6 Japan Research Institute of Artificial Joint, 725-1 Sugo, Kisarazu, Chiba 292-0036 Japan

**Keywords:** Outcomes research, Skeleton

## Abstract

The goal of this study was to investigate the impact of postoperative inclination of the joint line on clinical results after total knee arthroplasty (TKA) using a prosthesis with anatomical geometry. This study included 145 primary cruciate-retaining type of knee prosthesis with anatomical geometry. Three years postoperatively, clinical outcomes including the patient-reported outcomes (PROs) were recorded. Limb alignment was evaluated by the hip-knee-ankle (HKA) axis and inclination of the joint line was assessed by the joint line orientation angle (JLOA). Knees were divided into two groups according to the HKA: in-range (− 3 to 3°) and outlier group (< − 3° or > 3°) or the JLOA: in-range (2–4°) and outlier group (< 2° or > 4°), and clinical outcomes were compared between the groups. Postoperative Knee Society Function Score (KS-FS) was significantly higher in the HKA in-range group than the outlier group (*p* = 0.01). The Knee Society Knee Score and all subscales of the Knee injury Osteoarthritis Outcome Score were comparable between the groups. A multivariate analysis revealed a significant association between age at operation and postoperative KS-FS > of 80 points. Neither HKA in-range nor JLOA in-range were associated with the higher knee function. In conclusion, TKA-postoperative inclination of the joint line was not relevant to the short-term PROs. Treatment strategies that attempt to make joint line inclination in order to improve postoperative PROs should be avoided, and alignment goals such as kinematic alignment should be considered carefully.

## Introduction

Although the outcomes of total knee arthroplasty (TKA) are generally acceptable, approximately 20% of patients have some complaints and poor patient-reported outcomes (PROs) after TKA^1–3^. The reasons for dissatisfaction remain poorly understood. Failure to restore a physiological joint line has been suggested as a causative factor associated with the poor outcomes. In 2008, kinematically aligned (KA)-TKA was introduced as a surgical technique to reproduce the physiological joint line^4^. With KA-TKA, the femoral and tibial components are implanted to restore the physiological joint line to its constitutional state of individual patients. To date, many investigators have reported good clinical outcomes after KA-TKA^5–10^; however, the longevity of the polyethylene insert and survival of femoral and/or tibial components are still major concerns^11,12^. Considering these issues, restoration of the physiological joint line using a prosthesis with anatomical geometry through mechanically aligned (MA)-TKA may improve the PROs with maintaining the long-term survival.

The FINE total knee was developed in Japan and its clinical use began in 2001. It has unique design features that include an oblique 3° femorotibial joint line in both coronal and axial planes, conforming to anatomical geometry^13^. This feature allows reproduction of the physiological joint line by the osteotomy to be performed perpendicular to the mechanical axis. The patellofemoral geometry has patellar-friendly design and the round, all-polyethylene component is inset to the patella when resurfacing. When the FINE knee is implanted with mechanically neutral alignment, the forces on the insert should be even between the medial and lateral side. Besides, vitamin E is added to the polyethylene and the insert is produced through the direct compression molding method that allows to reduce backside wear of the polyethylene insert^14^. The insert has a minimum thickness of 5 mm on medial concave side that secures longevity of the implant.

Although restoration of the physiological joint line using a prosthesis with anatomical geometry would potentially improve the clinical outcomes including the PROs, the impact is not clear. The aim of this study was to investigate the impact of inclination of the joint line on clinical outcomes including PROs after TKA using a prosthesis with anatomical geometry. Our hypothesis was that patients with physiological inclination of the joint line after TKA would have better clinical outcomes including PROs than those without it.

## Materials and methods

### Patients

**Figure 1:**
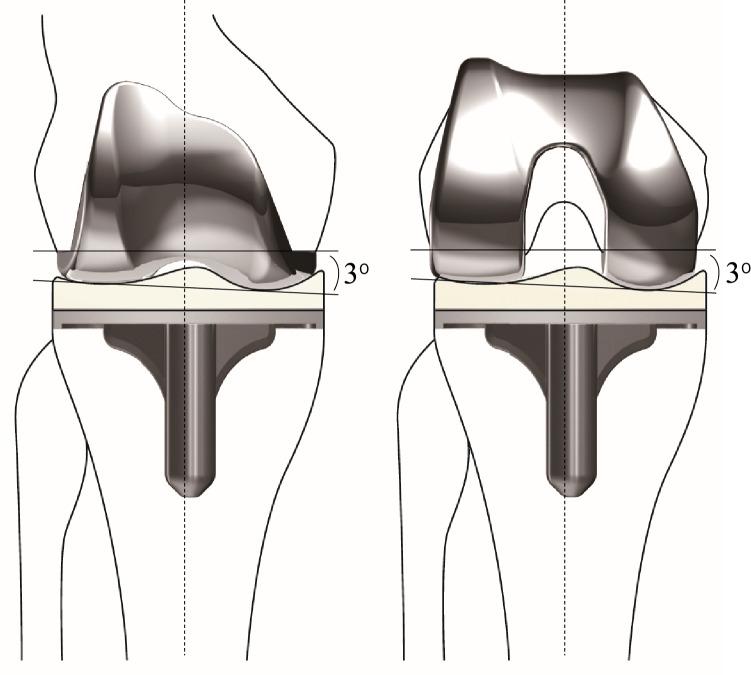
The FINE total knee. The femoral condyle has an asymmetric shape and femorotibial joint line with an inclination of 3° both in coronal (left) and axial (right) planes. The medial and lateral condyle have different length of radii (medial larger than lateral), resulting in medially inclined joint line both in extension and flexion. The medial surface of the polyethylene insert has a convex curve while the lateral surface has a flat surface. The figure is reprinted with minor modifications from Fig. 1 in reference 13.

This study included 145 primary TKAs in 145 patients with osteoarthritis (OA) who received a cruciate-retaining type of the FINE total knee (Teijin-Nakashima Medical Co. Ltd., Okayama, Japan) (Fig. 1) at our hospital between February 2015 and February 2019. Exclusion criteria were patients with an impaired posterior cruciate ligament, bilateral TKAs, valgus knees < 170° of the femorotibial angle (FTA), flexion contracture of 30^o^ or more, deformed femur or tibia following trauma, or dementia at the time of recording clinical scores.

This study was approved by the institutional review board at our institution. All activities were performed in accordance with the ethical standards set forth in the Declaration of Helsinki, and a written informed consent was obtained from all patients who participated in this study.

### Surgical procedures

All TKAs were performed by a single senior orthopedic surgeon (A.N) using the measured resection technique through the mid-vastus approach. Our goal was mechanically neutral alignment and surgeries were performed using conventional instruments. A distal femoral osteotomy was conducted perpendicular to the mechanical axis at a level 9–10 mm from the farthest point of the medial condyle, and the posterior condyle was osteotomized parallel to the surgical epicondylar axis. A tibial osteotomy subsequently was conducted perpendicular to the anatomical axis of the tibia. The resection level was 8–10 mm distal to the convex of the lateral plateau. Following osteotomy, soft tissue balance was checked both in extension and flexion using the knee balancer. When the minimum gap to implant the prosthesis was not obtained in medial side, the MCL was released from the tibial site or the pie-crust release was added. After no flexion contracture and good flexion angle in replacement with trial components was confirmed, the implants were fixed to the bone with cement. We basically replaced patellae but not for patients without osteoarthritic changes in patellofemoral joint or with small size of patellae. Patients were discharged three weeks after surgery when they were medically stable, with pain controlled by oral analgesics and when deemed by a physiotherapist to be mobilizing sufficiently to function safely at home.

### Clinical evaluation

Clinical scores including flexion angle, Knee Society Knee Score (KSS), and Knee injury and Osteoarthritis Outcome Score (KOOS) were recorded preoperatively and three years postoperatively. Flexion angle was measured by a single observer (M.Y) using a goniometer with a patient laid on a flat table, and the maximum active flexion angle was recorded. Increase in the scores (postoperative-preoperative scores) also were recorded. The KSS, which consists of a knee score (KS-KS) and a function score (KS-FS), was used to objectively evaluate knee function^15^. In addition to the KSS, the Japanese KOOS, an instrument of confirmed validity and reliability for PROs based on its cross-cultural adaptation^16^, was used, but the sports subscale was not recorded.

### Radiographic examinations for limb alignment and inclination of the joint line

**Figure 2:**
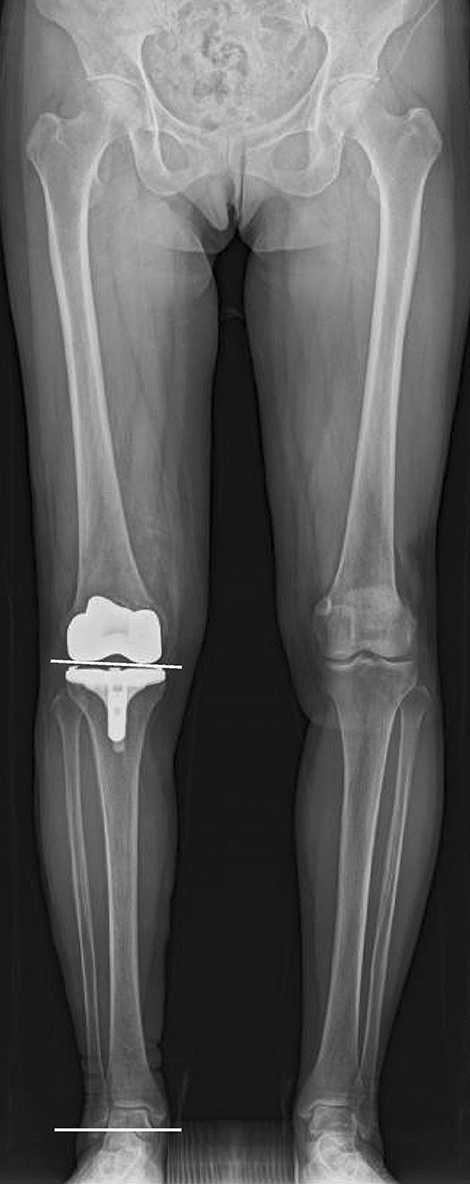
Joint line orientation angle (JLOA). The JLOA is the angle between the joint line and a line parallel to the floor, and medial inclination (medial side down and lateral side up) was defined as positive.

The limb alignment was evaluated by hip-knee-ankle (HKA) angle, which was measured using full-leg standing radiographs, and varus alignment was defined as positive. Knees were divided into two groups with the HKA: i.e., in-range (− 3 ~ 3°) and outlier groups (< − 3° or > 3°). Inclination of the joint line was evaluated by joint line orientation angle (JLOA) (Fig. 2), which is the angle formed between the joint line and a line parallel to the floor^9,17–20^. Medial inclination (medial side down and lateral side up) was defined as positive. The goal of the JLOA is considered to be 3° in the FINE based on its design concepts^13^. Therefore, knees were divided into two groups with the JLOA: i.e., in-range (2–4°) and outlier (< 2° or > 4°), and clinical outcomes were compared between the groups. Regarding the component alignment for individuals, our previous study has shown that there were no differences in coronal and sagittal alignment of the femoral and tibial components between the groups^21^.

Two independent observers (M.Y and A.N) measured the HKA and the JLOA twice, with a 2-month interval, using a subset of 20 cases. All measurements were performed using SYNAPSE-PACS software (FUJIFILM, Tokyo, Japan).

### Statistical analysis

Intra-observer and inter-observer reliabilities were assessed using intra-class correlation coefficients (ICCs). ICCs were > 0.8 for all measurements. Based on these reliabilities, measurements performed by a single investigator (M.Y) were used in the analysis. A *t*-test was used to compare between the two groups. Prior to the t-test, we confirmed that each clinical score exhibited a normal distribution and there were no significant differences in the variance between the groups. A statistical power analysis was performed prior the study; based on a prespecified significance level of α < 0.05 and by assuming a medium effect size (= 0.5), the power required was estimated to be 0.8 by using G*Power 3. The estimated sample size was 64 patients. A multivariate logistic regression analysis was further performed to determine factors associated with postoperative KS-FS > of 80 points. Age, gender (male/female), BMI, Kellgren-Lawrence (K-L) grade, preoperative range of motion (ROM), postoperative ROM, preoperative femorotibial angle (FTA), postoperative FTA, patellar replacement (yes/no), HKA in-range (yes/no), and JLOA in-range (yes/no) were chosen as the explanatory variables. Prior to the multivariate analysis, multi-collinearity for the variables was validated, and we confirmed that variance inflation factor (VIF) of each variable was < 10. Results were expressed as the mean (standard deviation, SD). Data analyses were performed using SPSS software, version 28 (SPSS Inc., Chicago, IL, USA) and *p*-values < 0.05 were considered statistically significant.

### Ethics approval and consent to participate

Approval for the study was received from the Institutional Review Board at Toho University Sakura Medical Center, and all patients gave their written consent to participate in this study. All activities were performed in accordance with the ethical standards set forth in the Declaration of Helsinki.

## Results

### Demographic data of the patients

The demographic data of the patients were shown in Table 1. There were 28 men and 117 women, with a mean age of 72.9 years (range: 48–89) at the time of surgery. The mean body mass index was 26.7 kg/m^2^. The mean FTA on a standing position was 184.4° (range: 172–206). The number of cases on the K-L classification^22^ were 7 for grade II, 29 for grade III, and 109 for grade IV. Ninety-seven patients (63.4%) received patellar replacement. Regardless of patellar replacement, neither anterior knee pain nor patellar clunk syndrome were observed during the period of this study.

**Table 1:** Summary of demographic data for patients included in this study.

Mean age at surgery, yrs (range)	72.9 (48–89)
Gender, female/male	117/28
Mean BMI at surgery (range)	26.7 (17.1–39.6)
Mean preoperative FTA (range)	184.4 (172–206)
Kellgren-Lawrence classification II/III/IV	7/29/109

BMI, body mass index; FTA, femorotibial angle.

### Distribution of the knees according to postoperative limb alignment or inclination of the joint line

**Figure 3:**
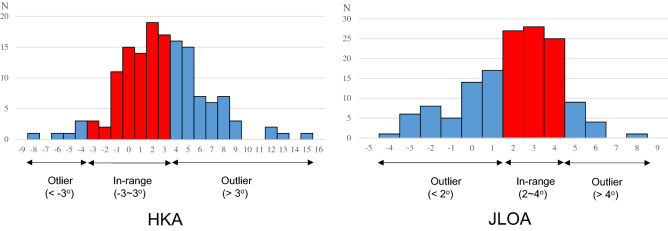
Distribution of the in-range and the outlier groups in a total of 145 knees. When the knees were classified according to the HKA axis, 81 knees (55.9%) were assigned to the in-range group (− 3 to 3°) (left, red bars), and 64 knees (44.1%) were assigned to the outlier group (< − 3° or > 3°) (left, blue bars). When classified according to the JLOA, 80 knees (55.2%) were assigned to the in-range group (2–4°) (right, red bars), and 65 knees (44.8%) were assigned to the outlier group (< 2° or > 4°) (right, blue bars). HKA: hip-knee-ankle; JLOA: joint line orientation angle.

Among 145 knees analyzed, 81 knees (55.9%) were classified into the HKA in-range group and 64 knees (44.1%) were classified into the outlier group. Alternatively, 80 knees (55.2%) were classified into the JLOA in-range group and 65 knees (44.8%) were classified into the outlier group. The distribution of knees either with the HKA or the JLOA is shown in Fig. 3.

### Comparison of clinical outcomes between groups for postoperative limb alignment

Preoperative flexion angle, the KSS, and all subscales of the KOOS were comparable between the HKA in-range and outlier groups. Postoperatively, the KS-FS was significantly higher in the in-range group than the outlier group (*p* = 0.01). The KS-KS, all subscales of the KOOS, and the increase in the scores were comparable between the groups. (Table 2).

**Table 2:** Comparison of clinical scores and patient-reported outcomes between groups for the HKA.

		In-range (n = 81)	Outlier (n = 64 )	95% CI difference	*p*-value
Flexion angle	Preoperative (SD)	118.9 (15.5)	119.2 (14.8)	− 4.685 to 5.345	0.897
	Postoperative (SD)	126.4 (11.0)	127.3 (10.7)	− 2.676 to 4.524	0.613
	Increase (SD)	7.5 (15.7)	8.1 (12.6)	− 4.165 to 5.353	0.805
KSS-KS	Preoperative (SD)	46.2 (16.1)	42.5 (15.0)	− 1.611 to 8.869	0.173
	Postoperative (SD)	95.8 (6.9)	96.7 (5.4)	− 1.216 to 2.949	0.412
	Increase (SD)	49.4 (18.3)	54.1 (15.0)	− 1.030 to 10.326	0.108
KSS-FS	Preoperative (SD)	42.9 (18.0)	38.0 (20.4)	− 1.493 to 11.357	0.131
	Postoperative (SD)	77.1 (18.2)	68.0 (24.0)	2.214 to 16.046	0.010*
	Increase (SD)	33.9 (22.5)	29.8 (23.6)	− 3.555 to 11.902	0.288
KOOS-symptom	Preoperative (SD)	50.7 (17.8)	46.9 (19.5)	− 2.316 to 9.973	0.220
	Postoperative (SD)	85.3 (12.6)	82.1 (17.8)	− 1.760 to 8.221	0.203
	Increase (SD)	34.6 (19.6)	35.2 (24.7)	− 6.677 to 7.874	0.871
KOOS-pain	Preoperative (SD)	45.1 (18.3)	44.5 (16.7)	− 5.300 to 6.381	0.855
	Postoperative (SD)	88.9 (12.3)	89.5 (11.6)	− 3.265 to 4.655	0.729
	Increase (SD)	44.2 (19.3)	45.0 (17.1)	− 5.261 to 6.911	0.789
KOOS-ADL	Preoperative (SD)	60.1 (15.6)	58.3 (16.5)	− 3.465 to 7.138	0.495
	Postoperative (SD)	85.8 (13.0)	84.4 (13.3)	− 3.011 to 5.667	0.546
	Increase (SD)	25.7 (17.0)	26.2 (19.1)	− 5.431 to 6.448	0.866
KOOS-QOL	Preoperative (SD)	27.8 (16.3)	24.1 (15.1)	− 1.564 to 8.883	0.168
	Postoperative (SD)	70.4 (19.3)	66.3 (21.8)	− 2.617 to 10.881	0.228
	Increase (SD)	42.7 (22.2)	42.2 (24.8)	− 7.265 to 8.211	0.904

HKA, hip-knee-ankle; KSS, Knee Society Score; KS, Knee Score; FS, Function Score; KOOS, Knee injury Osteoarthritis Outcome Score; SD, standard deviation; CI, confidence interval. *Significant difference, *p* < 0.05.

### Comparison of clinical outcomes between groups for postoperative inclination of the joint line

Preoperative KSS and the KOOS subscales were comparable between JLOA in-range and outlier groups, but the preoperative flexion angle was significantly lower in the in-range group than the outlier. The postoperative flexion angle was comparable between the groups (in-range: 126.6°, outlier: 127.1°). The increase in flexion angle was more in the in-range group than the outlier. The KSS, all subscales of the KOOS, and the increase in the scores other than flexion angle were comparable between the groups (Table 3).

**Table 3:** Comparison of clinical and patient-reported outcomes between groups for the JLOA.

		In-range (n = 80)	Outlier (n = 65)	95% CI difference	*p*-value
Flexion angle	Preoperative (SD)	116.4 (17.0)	122.3 (11.7)	1.022 to 10.843	0.018*
	Postoperative (SD)	126.6 (10.4)	127.1 (11.5)	− 3.145 to 4.049	0.804
	Increase (SD)	10.3 (15.2)	4.8 (12.7)	0.815 to 10.146	0.022*
KSS-KS	Preoperative (SD)	42.5 (14.7)	47.2 (16.5)	− 0.547 to 9.920	0.079
	Postoperative (SD)	96.1 (5.7)	96.2 (7.0)	− 2.078 to 2.090	0.996
	Increase (SD)	53.6 (15.4)	48.8 (18.7)	− 0.813 to 10.571	0.092
KSS-FS	Preoperative (SD)	41.4 (18.1)	39.8 (20.7)	− 4.913 to 8.076	0.631
	Postoperative (SD)	75.6 (17.9)	69.9 (24.7)	− 1.303 to 12.706	0.110
	Increase (SD)	34.0 (20.0)	29.5 (26.4)	− 3.232 to 12.266	0.251
KOOS-symptom	Preoperative (SD)	49.0 (18.1)	49.0 (19.4)	− 6.165 to 6.171	0.999
	Postoperative (SD)	84.7 (15.9)	82.9 (14.2)	− 3.207 to 6.797	0.479
	Increase (SD)	35.7 (20.4)	33.9 (23.8)	− 5.461 to 9.057	0.625
KOOS-pain	Preoperative (SD)	46.0 (15.6)	43.4 (19.7)	− 3.235 to 8.399	0.382
	Postoperative (SD)	90.1 (11.3)	88.0 (12.7)	− 1.868 to 6.013	0.300
	Increase (SD)	44.5 (15.0)	44.6 (21.7)	− 6.000 to 6.157	0.980
KOOS-ADL	Preoperative (SD)	59.8 (14.8)	58.5 (17.4)	− 3.820 to 6.772	0.583
	Postoperative (SD)	86.7 (11.7)	83.3 (14.5)	− 0.909 to 7.694	0.121
	Increase (SD)	26.8 (15.9)	24.8 (20.2)	− 4.006 to 7.839	0.523
KOOS-QOL	Preoperative (SD)	26.4 (15.0)	25.9 (16.9)	− 4.748 to 5.750	0.851
	Postoperative (SD)	70.9 (21.0)	65.8 (19.5)	− 1.579 to 11.860	0.133
	Increase (SD)	44.6 (20.5)	39.9 (26.3)	− 3.049 to 12.328	0.235

JLOA, joint line orientation angle; KSS, Knee Society Score; KS, Knee Score; FS, Function Score; KOOS, Knee injury Osteoarthritis Outcome Score; SD, standard deviation; CI, confidence interval. *Significant difference, *p* < 0.05.

### A multivariate analysis for identification of factor(s) associated with the postoperative KS-FS > of 80 points

Based on the results that the in-rage group for the HKA had a significantly higher score than the outlier group (Table 2), we performed a multivariate logistic analysis to identify factor(s) associated with the postoperative KS-FS > of 80 points. Among 11 explanatory variables, age at operation was significantly associated with the postoperative KS-FS > of 80 points, and neither HKA in-range nor JLOA in-range were associated with the higher knee function (Table 4).

**Table 4:** A multivariate analysis for the factors associated with postoperative KS-FS > of 80 points.

	Odds ratio	95% CI	*p*-value
Gender (female/male)	0.363	0.116–1.140	0.083
Age at operation	0.857	0.800–0.919	< 0.001*
BMI	0.996	0.895–1.109	0.942
Patellar replacement (yes/no)	0.287	0.265–1.481	0.287
Preoperative ROM	1.013	0.991–1.035	0.267
Postoperative ROM	1.022	0.983–1.063	0.278
Preoperative FTA	0.975	0.890–1.069	0.588
Postoperative FTA	1.004	0.827–1.219	0.967
K-L grade	0.709	0.292–1.724	0.448
HKA in-range (yes/no)	0.818	0.305–2.195	0.690
JLOA in-range (yes/no)	0.706	0.301–1.655	0.423

KS-FS, Knee Society Function Score; BMI, body mass index; ROM, range of motion; FTA, femorotibial angle; K-L, Kellgren-Lawrence; HKA, hip-knee-ankle axis; JLOA, joint line orientation angle; CI, confidence interval. *Significant difference, *p* < 0.05.

## Discussion

The association between postoperative mechanical axis alignment, often evaluated as HKA axis alignment, and clinical outcomes after mechanically aligned (MA)-TKA has been shown by a number of studies. Most studies stated that HKA axis malalignment was not associated with clinical outcomes^13,23–28^. Similar results were shown in the present study; the HKA in-range group did not have better clinical outcomes including flexion angle, the KSS, and all subscales of the KOOS than the outlier group. However, long-term clinical outcomes and implant survival are concerned in TKA with HKA axis malalignment. Kuroda et al. reported that immediate postoperative varus alignment and varus position of the femoral and tibial components were possible risk factors for varus progression in limb alignment in the long term after TKA^29^. Therefore, it is conceivable that the postoperative HKA axis should be targeted for in-range alignment even though it does not associate with clinical outcomes.

Inclination of the joint line after TKA has been discussed by many orthopedic surgeons. Several investigators have examined physiological inclination of the joint line in normal knees^30–36^. Moreland et al. first reported that inclination of the joint line relative to the tibial mechanical axis was 3.0° (right) and 2.6° (left) in young male volunteers ^30^. Nakano et al. reported that tibial plateau inclination was 85.6° (female) and 85.1° (male) in young Japanese participants^36^, which corresponds to 4.4° and 4.9° respectively, if inclination of the joint line was measured according to the method in the present study. These observations show physiological inclination of the joint line in normal knees and its racial or gender differences. Regarding the joint line inclination in OA knees, we have shown that the mean medial proximal tibial angle (MPTA) for elderly Japanese patients was 83.9°^37^, which corresponds to 6.1 degrees of inclination in the present study.

The FINE total knee was developed to reproduce physiological inclination of the joint line. The characteristic aspect of the FINE knee is to have 3° inclination of the medial femorotibial surface and anatomical geometry^13^. The expectation using this prosthesis was that patients with a JLOA close to 3° would have better clinical outcomes than those with a JLOA that deviated from 3°. A systematic review showed that the clinical outcomes of KA-TKA were comparable or superior to those of MA-TKA after a minimum 2-year follow-up. This review also showed that the JLOA in KA-TKA was relatively parallel to the floor compared to that in the native knee, and not oblique (medial side up and lateral side down) compared to that in MA-TKA^9^. In the present study, 55.2% of knees were classified to the in-range group with the JLOA, and 44.8% were classified to the outlier group. Although the goal of JLOA in the FINE knee is considered to be 3°, the in-range group did not have better clinical results including flexion angle, the KSS, and all subscales of the KOOS than the outlier group. Similar results have been shown by previous studies^5–10^. In this study, we showed that a half of patients got ‘in-range’ JLOA when the ‘in-range’ was defined between 2 and 4° with such limited range (Fig. 3); however, this does not mean that there are only 50% chances to get good alignment when performing TKA using our surgical technique. It remains to be argued whether the JLOA of 2–4° is adopted to ‘in-range’.

A recent classification for coronal plane alignment of the knee (CPAK) has simplified categorization of knee phenotypes based on two independent variables, constitutional (prearthritic) alignment, termed the arithmetic HKA, and joint line obliquity^38^. Sappey-Marinier et al., using the CPAK classification, investigated whether better clinical outcomes would be obtained in patients with a restored knee phenotype at 2 years follow-up after MA-TKA^39^. The results showed that only 18% of patients had restored constitutional knee phenotype, and neither restored obliquity nor arithmetic HKA was associated with clinical outcomes including flexion angle and the KSS. These observations suggest that performing MA-TKA resulted in most cases in a change of the preoperative knee phenotype, and restoration of the knee phenotype did not always lead to better clinical outcomes. As the clinical outcomes were short-term and physician-based alone, the mid- to long-term follow-up and the PROs will be required to evaluate the importance of restoration of the knee phenotype. The femoral rotational alignment is also likely to change when performing TKA in the concept to restore preoperative joint line inclination. The impact of postoperative inclination of the joint line in the femoral axial plane on clinical outcomes should be investigated in the future studies.

The multivariate logistic analysis revealed significant association between age at operation and postoperative KS-FS of > 80 points, and neither HKA in-range nor JLOA in-range were associated with the higher function (Table 4). These results demonstrate that younger age at surgery is important to obtain higher knee function and may suggest the alignment or joint line inclination to be targeted should not be limited in the narrow range regarding functional outcome in the short-term.

Recently, a wide distribution of femoral and tibial coronal alignment in normal knees has been identified^40–44^, which suggests the need for a more individualized approach in TKA^40^. Taken together with our results, reconstructing inclination of the joint line to a uniform angle, that is 3°, may not lead to an improvement in clinical outcomes. It might be a better surgical strategy to reproduce the inclination of the joint line to its constitutional state in each patient. Further investigation with mid- to long-term follow-ups will be required to demonstrate the impact of restoration of inclination of the joint line on clinical outcomes after TKA.

In this study we replaced patellae for 63.4% of patients, which might affect clinical outcomes including PROs. However, in the multivariate analysis patellar replacement was not associated with higher knee function (Table 4). A long-term follow-up will be necessary to evaluate the influence of patellar replacement on clinical outcomes.

There are some limitations to this study. First, the sample size was relatively small and the patients were recruited from a single institution. To establish the impact of postoperative joint line on clinical outcomes, more cases will be required from multiple institutions. Second, approximately 40% of patients did not receive patellar replacement, which could not be ignored when investigating the influence of other variables on postoperative outcomes. Third, inclination of the joint line was assessed by a coronal plane alone, and it should be also assessed by a sagittal plane in the future studies. Fourth, knees were divided into two groups (the HKA or JLOA in-range and outlier). Especially, it remains to be argued to set physiological inclination of the joint line at 2–4° of the JLOA.

In conclusion, neither TKA-postoperative inclination of the joint line nor limb alignment was relevant to the short-term PROs. Treatment strategies that attempt to make joint line inclination in order to improve postoperative PROs should be avoided, and alignment goals such as kinematic alignment should be considered carefully. Further investigation with mid- to long-term follow-ups will be required to show the impact of restoration of physiological inclination of the joint line on clinical outcomes after TKA.

## Data Availability

The datasets used and/or analyzed during the current study are available from the corresponding author on reasonable request.
